# Complete mitochondrial genome of *Laudakia sacra* (Reptilia: Agamidae)

**DOI:** 10.1080/23802359.2019.1636724

**Published:** 2019-07-16

**Authors:** Zhang Yong, Li-Fang Peng, Peng-Hui Xu, Shuang-Quan Duan

**Affiliations:** a Institute of Biodiversity Science and Geobiology, College of Science, Tibet University, Lahsa, China;; b College of Life and Environment Sciences, Huangshan University, Huangshan, China

**Keywords:** Mitogenome, Agamidae, *Laudakia sacra*

## Abstract

The complete mitochondrial genome (mitogenome) sequence of *Laudakia sacra* was determined by using a PCR-based method. The total length of mitogenome is 16,555 bp, and contains 13 typical vertebrate protein-coding genes, 22 transfer RNA genes, 2 ribosomal RNA genes and 2 control regions. Only ND6 gene and 8 tRNA genes on the L-strand other than are encoded on the H-strand. The phylogenetic tree of *L. sacra* and 13 other species were built. The DNA data present here will facilitate future taxonomic work of the genus *Laudakia*.

The genus *Laudakia* (Gray, 1845) contains approximately 20 species (Zhao et al. 1999; Baig et al. 2012; Wang et al. 2014). *Laudakia sacra* is endemic to Tibetan Plateau, which distributed from the west of Mainling County to the east of Lhatse County along Yarlung Zangbo River. Here, we determined and described the complete mitogenome of *L. sacra* in order to obtain basic genetic information about this species, enrich the *Laudakia* species genome resource and promote further research concerning related species.

The specimen of *L. sacra* (Voucher number: HSR18121) was collected from Nyemo County, Xizang (Tibet) Autonomous Region, China (N29°21′18″, E90°9′1″; 3750 m). The tissue of the specimen was preserved at Museum of Huangshan University. The complete mitogenome of *L. sacra* (Genbank accession number MK411596) was sequenced to be 16,555 bp which contain 37 genes: 13 typical vertebrate protein-coding gene, 22 transfer RNA genes, 2 ribosomal RNA genes and 2 control regions (D-loops), which is similar to the typical vertebrate mtDNA (Boore 1999).

All the proteins of *L. sacra* were distributed on the H-strand, except for the ND6 subunit gene and 8 tRNA genes which were encoded on the L-strand. The positions of RNA genes were predicted by the MITOS (Bernt et al. 2013) and the locations of proteins were identified by comparing with the homologous genes of other closely related species. The overall base composition of the entire genome was as follows: A (36.4%), T (23.8%), C (27.4%) and G (12.5%), of which the percentage of A + T (60.2%) reflected a typical sequence feature of the vertebrate mitogenome. Among the mitochondrial proteins, the ATP8 was the shortest and the ND5 was the longest.

Nine of the 13 proteins (COI, COII, ATPase 8, ATPase 6, COIII, ND4L, ND4, ND5, and ND6) was initiated with ATG as start codon, while ND1 and ND2 genes start with ATA, and ND3 and CYT b initiate with ATT. Nine genes (ND1, ND2, COI, COII, ATPase 8, ND4L, ND4, ND5 and ND6) end with complete stop codons (TAG, AGA, AGG and TAA), and other four genes termination codon T.

The 22 tRNA genes range in size from 57 to 75 bp. The 12s rRNA (881 bp) and 16s rRNA (1498 bp), are located between the tRNA-Pro and tRNA-Leu gene and separated by the tRNA-Val gene. The D-loop 1 of the *L. sacra* mitogenome in size is 1032 bp and located between the tRNA-Pro and rRNA-Ser genes, and the D-loop 2 is 329 bp long, between the tRNA-Thr and tRNA-Phe genes.

In order to validate the new determined sequences, we selected the 12 protein-coding genes located on heavy strand except for ND6 which encoded on the light strand of *L. sacra* in this study, and together with other 13 related species from GeneBank to perform phylogenetic analysis. These species were as follows: *Laudakia sacra*, *L. tuberculate*, *Xenagama taylori*, *Pogona vitticeps*, *Chlamydosaurus kingii*, *Hydrosaurus amboinensis*, *Leiolepis belliana*, *Acanthosaura armata, Phrynocephalus sinaitus*, *P. albolineatus*, *P. grumgrzimailoi*, *P. frontalis*, *P. przewalskii* and *P. mystaceus*. A maximum likelihood (ML) tree was constructed based on the dataset by online tool RAxML (Kozlov et al. 2018) (Figure 1). The phylogenetic analysis result was consistent with the previous research (Pyron et al. 2013; Peng et al. 2019). It indicated that our new determined mitogenome sequences could meet the demands and explain some evolution issues.

**Figure 1.**
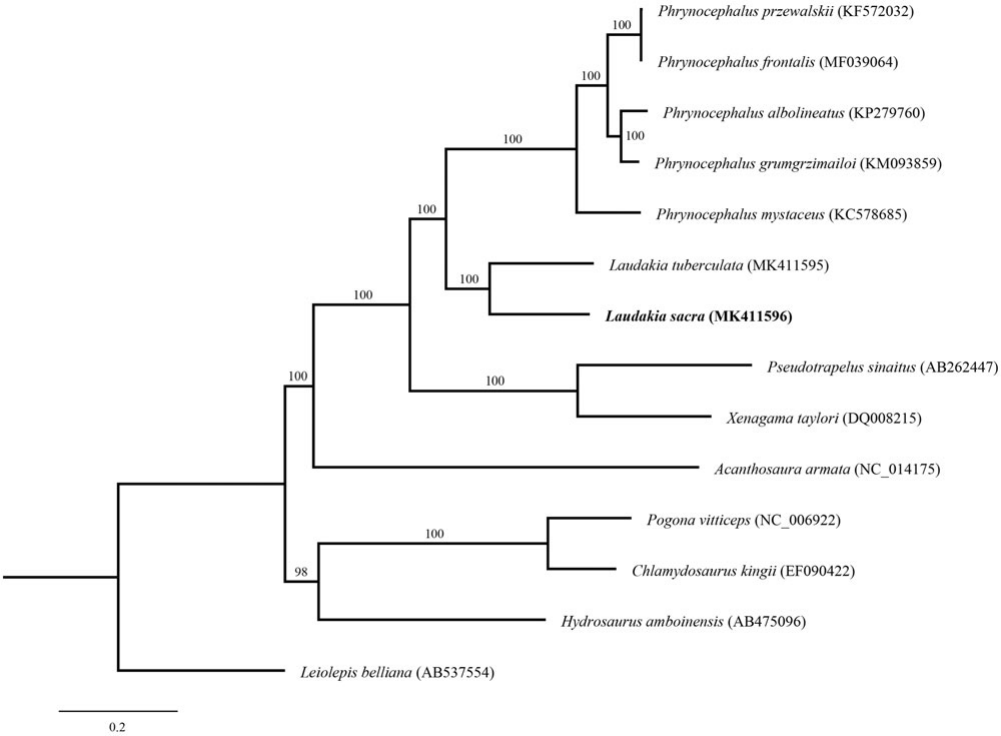
A maximum likelihood (ML) tree of Laudakia sacra in this study and other 13 related species was constructed based on the dataset of 12 concatenated mitochondrial protein-coding genes by online tool RAxML. The numbers above the branch meant bootstrap value. Bold black branches highlighted the study species and corresponding phylogenetic classification.
